# Ongoing Transmission of *Onchocerca volvulus* after 25 Years of Annual Ivermectin Mass Treatments in the Vina du Nord River Valley, in North Cameroon

**DOI:** 10.1371/journal.pntd.0004392

**Published:** 2016-02-29

**Authors:** Albert Eisenbarth, Mbunkah Daniel Achukwi, Alfons Renz

**Affiliations:** 1 Institute of Evolution and Ecology, Department of Comparative Zoology, University of Tübingen, Tübingen, Germany; 2 Programme Onchocercoses field station of the University of Tübingen, Ngaoundéré, Cameroon; 3 Veterinary research laboratory, Institute of Agricultural Research for Development, Wakwa Regional Centre, Ngaoundéré, Cameroon; Common Heritage Foundation, NIGERIA

## Abstract

**Background:**

Recent reports of transmission interruption of *Onchocerca volvulus*, the causing agent of river blindness, in former endemic foci in the Americas, and more recently in West and East Africa, raise the question whether elimination of this debilitating disease is underway after long-term treatment of the population at risk with ivermectin. The situation in Central Africa has not yet been clearly assessed.

**Methods and findings:**

Entomologic data from two former endemic river basins in North Cameroon were generated over a period of 43 and 48 months to follow-up transmission levels in areas under prolonged ivermectin control. Moreover, epidemiologic parameters of animal-borne *Onchocerca* spp. transmitted by the same local black fly vectors of the *Simulium damnosum* complex were recorded and their impact on *O*. *volvulus* transmission success evaluated. With mitochondrial DNA markers we unambiguously confirmed the presence of infective *O*. *volvulus* larvae in vectors from the Sudan savannah region (mean Annual Transmission Potential 2009–2012: 98, range 47–221), but not from the Adamawa highland region. Transmission rates of *O*. *ochengi*, a parasite of Zebu cattle, were high in both foci.

**Conclusions/significance:**

The high cattle livestock density in conjunction with the high transmission rates of the bovine filaria *O*. *ochengi* prevents the transmission of *O*. *volvulus* on the Adamawa plateau, whereas transmission in a former hyperendemic focus was markedly reduced, but not completely interrupted after 25 years of ivermectin control. This study may be helpful to gauge the impact of the presence of animal-filariae for *O*. *volvulus* transmission in terms of the growing human and livestock populations in sub-Saharan countries.

## Author Summary

Over the past decades the Fight against river blindness, a tropical disease caused by a nematode worm, has been relatively successful, and a number of countries have been reported to be free of parasite transmission. In North Cameroon, we checked the occurrence of infective stages of *Onchocerca volvulus* in the transmitting black fly populations for more than three years and were able to confirm that the transmission there is low, but not yet interrupted. In a second location on a highland plateau, however, no infective stages of the human parasite were found. Instead, a closely-related parasite of cattle was present in both places. Given that the areas are not far away from each other and the biting frequencies of the black fly populations are similar, the historically earlier and higher density of cattle herds in one of the regions would explain why it is now free of the parasite due to the effects called zooprophylaxis and cross-reacting premunition. Changes in the socio-economic environment, especially the increase of human and cattle populations have a strong influence on the spread of river blindness in Africa.

## Introduction

The interruption of transmission of *Onchocerca volvulus*, the causing agent of river blindness or onchocerciasis, has been confirmed for a growing number of endemic foci on the American continent [1,2,3] and in West [4] and East Africa [5,6,7]. The recent success in onchocerciasis control can be mainly attributed to the extensive and sustained mass treatment programs with the microfilaricide ivermectin, governed by institutions of the World Health Organization, like the African Programme for Onchocerciasis Control [8]. Long treatment rounds are necessary because the drug is only lethal to the larval stage and not the adult worm.

There are thus good prospects that elimination of onchocerciasis is well underway in the Americas [9] and may also have begun at different foci on the African continent [6,10,11]. However, currently there is a paucity of information on the actual situation in Central and Southern Africa, in particular with respect to vector transmission, albeit a significant proportion of these regions have been hyperendemic. Recent studies on the effects of ivermectin treatment on the epidemiology of *O*. *volvulus* in humans and the black fly vector *Simulium damnosum sensu lato* have been done in North and West Cameroon [12,13,14,15,16]. The caveat of the most recent studies is that the filarial species in the vector were not always correctly identified, and the prevalence of infective *O*. *volvulus* larvae and thus the transmission potential remains unknown. Local *S*. *damnosum s*.*l*. populations are vectors of at least two other species from the *Onchocerca* genus: *O*. *ochengi*, a common parasite of Zebu cattle *Bos indicus* [17] and *O*. *ramachandrini*, a filaria from the warthog *Phacochoerus africanus* [18]. The proportion of animal-filariae in the vector has direct and indirect consequences for parasite transmission to humans [19,20,21] rendering it an important factor to understand the epidemiology of river blindness. Furthermore, filariae closely-related to *O*. *volvulus* might repopulate the human host [22] posing a potential risk of infection, or they might transfer genes to *O*. *volvulus* which negatively affect the effectiveness of ivermectin, presently the sole drug intervention in use [23]. For this reason we combine microscopic differentiation of infective larvae with a PCR-based molecular approach which allows the detection of yet unknown filarial species and strains in addition to already known *Onchocerca* spp. [22].

This study presents the latest entomologic data of a longitudinal study in the Vina du Nord valley, North Cameroon, dating back to 1976 when ivermectin mass treatment had not yet commenced [24,25]. The impact on *O*. *volvulus* transmission after 25 years of annual community-directed treatment with ivermectin (CDTI) is demonstrated here. Furthermore, a second site endemic for onchocerciasis in an economically important cattle livestock region has been monitored since 1985. The epidemiologic data is also complemented with *Onchocerca* spp. transmitted by the same local vectors of the *S*. *damnosum* complex and discussed in light of their impact on transmission success of *O*. *volvulus*. We have not studied onchocerciasis transmission in regions where ivermectin treatment is contraindicated, such as co-endemic foci of *Loa loa* in the Central African rainforest, although they remain potential source-areas for reinvasion.

## Methods

### Study sites

**Fig 1:**
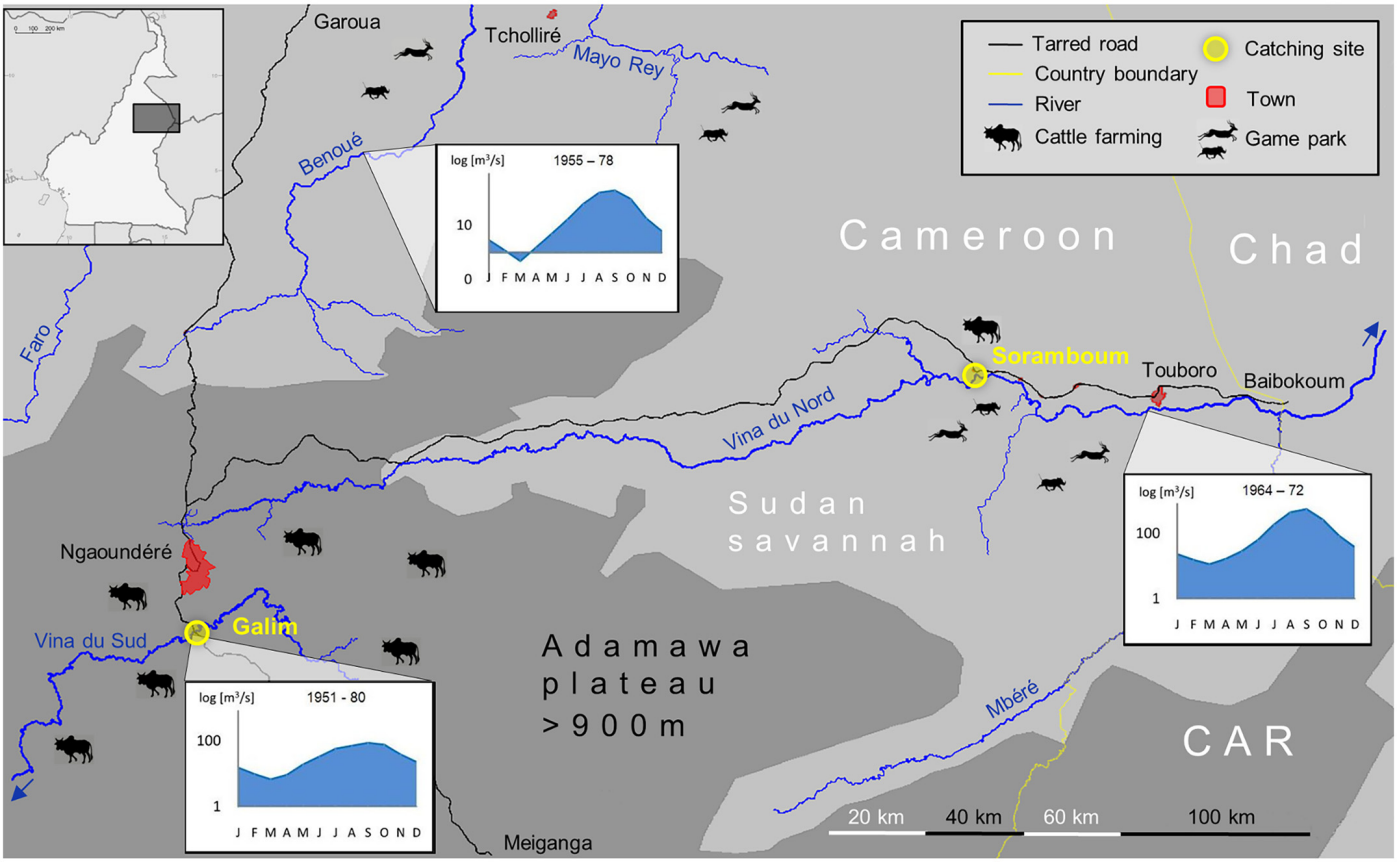
Study area. Overview map of the study sites (yellow circles) from two river basins in Northern Cameroon. The village Galim at the Vina du Sud river near Ngaoundéré is on the Adamawa highland plateau, an area above 900 m altitude (dark grey area) with intense cattle husbandry. The village Soramboum at the Vina du Nord river towards Touboro is located in the Sudan savannah (light grey area), a region not often frequented by cattle herds and with high wildlife density. Diagrams of hydrological data showing the mean monthly water discharge from three river basins is taken from Olivry [26]. CAR: Central African Republic.

Two *S*. *damnosum* fly catching sites at two foci in Northern Cameroon were visited between two to four times per month (Fig 1). One former hyperendemic onchocerciasis focus is the village Soramboum close to the Vina du Nord river in the Sudan savannah (500 m altitude): 7°47'14"N; 15°0'22"E where ivermectin mass treatments have been conducted since 1987. Here we present entomological data collected from September 2009 till March 2013. The village has approximately 1000 inhabitants today, and between 1000 and 2000 cattle are located in the vicinity (personal observation). The Vina du Nord river is flowing perennially with an average annual water discharge of 150 m^3^ per second, with highest values between July and October [26]. The other formerly hypo- to mesoendemic focus monitored is the village Galim located 15 km south of Ngaoundéré (population: 500 inhabitants, approximately 5000 cattle in the vicinity, personal observation) at the Vina du Sud river (mean annual water discharge: 37 m^3^/s, 1050 m altitude): 7°12'2"N; 13°34'56"E, where CDTI has started in 1997. The entomological data was collected from April 2009 till March 2013. The area belongs to the Guinea-grassland of the Adamawa plateau, located in an important area for cattle livestock production in Cameroon [27]. Baseline and follow-up data of *O*. *volvulus* transmission to man before ivermectin mass treatments started is available for both foci [15,16,19,25,28], and publicly available via the project website www.riverblindness.eu (http://riverblindness.eu/epidemiology/fly-catching-sites-data/). Whereas *S*. *damnosum sensu stricto* and *S*. *sirbanum* are the predominant vector species at the Vina du Nord river, it is *S*. *squamosum* at the Vina du Sud river [29].

Fly catching was performed according to Duke *et al*. [30] with the following modifications. Blood meal-searching female *Simulium* flies were attracted on man by exposing the fly catcher’s legs and trapped with sucking tubes as soon as they settled before starting to probe. Usually the catching period was from 7 am to 6 pm, interrupted by a one hour break at noon. Afterwards, the catches were transported to the research station in Ngaoundéré and stored at -15°C until dissection.

### Fly dissection and identification of filarial species

Flies were sorted, counted, and a maximum of 100 female *S*. *damnosum s*.*l*. flies per site and day were dissected with needles under a stereomicroscope (Wild M5, Switzerland). The parous rate was determined by examination of the ovaries in the abdomen [31]. From parous flies infested with filarial worms the location (head, thorax or abdomen), molting stage and quantity was noted. Following the identification key of Wahl *et al*. [15], infective third-stage larvae (L3) were classified to species according to body length, measured by an ocular eye-micrometer attached to the stereomicroscope at 50x magnification, and shape of the anterior and posterior ends. For a subdivision of the L3 taken between February 2010 and February 2012, a molecular investigation of their mitochondrial DNA was conducted according to Eisenbarth *et al*. [22]. Briefly, single L3’s were digested with 1 to 2 μl proteinase K (20 μg/μl stock) in 75 μl DirectPCR lysis reagent (Viagen Biotech, USA) at 55°C. Two μl of the crude extract was used for each 25 μl PCR reaction. Primer pairs of three mitochondrial loci (12S rRNA, 16S rRNA and *coxI)* that allow for the discrimination of filarial species were used. The amplified PCR products were sequenced on an ABI Prism 3100 genetic analyzer (Applied Biosystems, USA) following the manufacturer’s protocol. For the comparison of the body lengths of L3's identified by molecular markers, a larger sample size was taken from flies caught in the same period at the Vina du Sud river about one kilometer downstream of the site near Galim. These flies were collected both from man and cattle.

### Calculation of Annual Biting Rates and Transmission Potentials

The Annual Biting Rate (ABR) and Annual Transmission Potential (ATP) of *Simulium damnosum* flies were determined according to the literature [24,25,32]. First, the monthly biting rates (MBR) were calculated by the sum of the flies caught per month divided by the number of catching days and multiplied by the number of days per month. No correction was made for the missing hours due to rain, sandstorm, or any other reasons. The ABR is the sum of 12 MBRs per hydrological year, measured from April (beginning of rainy season) till March next year (end of dry season). For months during which no catches were attempted, the mean MBR value for the corresponding month and site over the respective decade was estimated by interpolation. The monthly infection rate was the sum of the infective L3 of *O*. *volvulus*, *O*. *ochengi* and *O*. *ramachandrini* from the head, thorax and abdomen found in all parous flies, divided by the sum of dissected flies. By multiplying the monthly infection rate with the respective MBR, the Monthly Transmission Potential (MTP) was determined. The ATP is the sum of 12 MTPs for one year, starting from April till March next year. Missing data points were extrapolated by the sum of all MTP divided by the number of months with data, and multiplied by factor 12. If the MTP data per year was below 3, the mean annual infection rate of proximate years multiplied with the respective ABR was used instead for the ATP calculation.

### Statistical analysis

The statistical program Python version 3.4.1 was used for statistical analysis employing student *t*-tests. Results were considered statistically significant when the p-value was below 0.05. P-values were corrected for multiple testing by multiplying with the number of tests done. The effect size was calculated according to Cohen [33]. For depicting the distribution of the L3 body length from a random sample, violin plots, *i*.*e*. box plots with a rotated kernel density plot on each side, were used.

## Results

### Entomological data

During the study period, a total of 39,082 flies were caught on human fly catchers, and 21,897 (55.6%) of them were dissected: a total of 2096 L3 were found (Table 1). Depending on the catching site, a mean of 1.96 ±0.53 L3 (max. 20) were harvested per infective fly in Soramboum and a mean of 3.49 ±0.13 L3 (max. 23) in Galim. Near Soramboum at the Vina du Nord site the infection rate (flies carrying L1, L2 and L3) of parous flies in the rainy season was significantly higher than in the dry season (mean: 10.9 *vs*. 5.9%, *t*-value = -4.91, p < 0.001), as well as the infection rate with infective L3 stages (mean: 7.8 *vs*. 3.3%, *t*-value = -4.88, p < 0.001). The opposite was true at the Vina du Sud site near Galim (mean infection rate: 11.1 *vs*. 16.6%, *t*-value = 2.36, p > 0.05; mean L3 infection rate: 4.3 *vs*. 7.1%, *t*-value = 1.89, p > 0.05). Moreover, the parous rate, which is a parameter of age structure, was on average 9.6% higher in the wet season than in the dry season in Soramboum (range: -18.3–19.3%, t-value = 5.31, p < 0.001), but in Galim on average 11.8% lower in the wet season compared to the dry season (range: 6.7–24.3%, t-value = 3.83, p < 0.001). The higher proportion of infective L3 to developing larvae during the rainy season in Soramboum (64.0% *vs*. 53.5%, t-value = -1.88, p > 0.05), but not in Galim (33.9% *vs*. 35.3%, t-value = 0.88, p > 0.05) is lacking statistical support. A generally higher proportion of L3 in Soramboum can be explained by a longer storage time of the caught flies at ambient temperature during the time (often days) until they were brought to the laboratory, 225 km away by public transport, so that more larval stages developed further.

**Table 1:** Entomologic parameters and proportion of filarial stages from *Simulium damnosum s*.*l*. at two different sites in Northern Cameroon.

Location		RS 2009		DS 2009/10		RS 2010		DS 2010/11		RS 2011		DS 2011/12		RS 2012		DS 2012/13		Total RS		Total DS	
Soramboum/ Vina du Nord	flies caught	84		5.776		11.403		3.692		1.613		1.344		561		772		13.661		11.584	
	dissected (*%*^a^)	84	*100*,*0*	2.141	*37*,*1*	4.236	*37*,*1*	2.636	*71*,*4*	1.393	*86*,*4*	1.128	*83*,*9*	561	*100*,*0*	772	*100*,*0*	6.274	*45*,*9*	6.677	*57*,*6*
	parous (*%*^b^)	34	*40*,*5*	1.067	*49*,*8*	2.687	*63*,*4*	1.844	*70*,*0*	1.216	*87*,*3*	829	*73*,*5*	437	*77*,*9*	504	*65*,*3*	4.374	*69*,*7*	4.244	*63*,*6*
	infected (*%*^c^)	4	*11*,*8*	84	*7*,*9*	303	*11*,*3*	101	*5*,*5*	128	*10*,*5*	45	*5*,*4*	40	*9*,*2*	19	*3*,*8*	475	*10*,*9*	249	*5*,*9*
	infective (*%*^d^)	3	*8*,*8*	44	*4*,*1*	234	*8*,*7*	59	*3*,*2*	77	*6*,*3*	24	*2*,*9*	25	*5*,*7*	11	*2*,*2*	339	*7*,*8*	138	*3*,*3*
	L1 (*%*^e^)	0	*0*,*0*	30	*14*,*6*	54	*8*,*4*	52	*28*,*6*	47	*16*,*5*	40	*34*,*5*	8	*9*,*6*	6	*24*,*0*	109	*10*,*7*	128	*24*,*2*
	L2 (*%*^e^)	1	*20*,*0*	73	*35*,*4*	156	*24*,*2*	24	*13*,*2*	80	*28*,*1*	19	*16*,*4*	20	*24*,*1*	2	*8*,*0*	257	*25*,*3*	118	*22*,*3*
	L3 (*%*^e^)	4	*80*,*0*	103	*50*,*0*	434	*67*,*4*	106	*58*,*2*	158	*55*,*4*	57	*49*,*1*	55	*66*,*3*	17	*68*,*0*	651	*64*,*0*	283	*53*,*5*
Galim/ Vina du Sud	flies caught	812		3.069		987		1.660		1.379		4.402		813		715		3.991		9.846	
	dissected (*%*^a^)	757	*93*,*2*	1.783	*58*,*1*	947	*95*,*9*	1.014	*61*,*1*	962	*69*,*8*	2.168	*49*,*3*	773	*95*,*1*	542	*75*,*8*	3.439	*86*,*2*	5.507	*55*,*5*
	parous (*%*^b^)	407	*53*,*8*	1.049	*58*,*8*	515	*54*,*4*	645	*63*,*6*	601	*62*,*5*	1.453	*67*,*0*	422	*54*,*6*	391	*72*,*1*	1.945	*56*,*6*	3.538	*64*,*2*
	infected (*%*^c^)	35	*8*,*6*	199	*19*,*0*	73	*14*,*2*	114	*17*,*7*	72	*12*,*0*	225	*15*,*5*	35	*8*,*3*	50	*12*,*8*	215	*11*,*1*	588	*16*,*6*
	infective (*%*^d^)	12	*2*,*9*	86	*8*,*2*	44	*8*,*5*	53	*8*,*2*	19	*3*,*2*	84	*5*,*8*	8	*1*,*9*	27	*6*,*9*	83	*4*,*3*	250	*7*,*1*
	L1 (*%*^e^)	54	*45*,*8*	408	*40*,*6*	28	*12*,*3*	146	*35*,*8*	109	*39*,*6*	402	*42*,*0*	62	*48*,*4*	59	*29*,*4*	253	*33*,*8*	1.015	*39*,*5*
	L2 (*%*^e^)	32	*27*,*1*	219	*21*,*8*	78	*34*,*2*	108	*26*,*5*	98	*35*,*6*	272	*28*,*4*	34	*26*,*6*	49	*24*,*4*	242	*32*,*3*	648	*25*,*2*
	L3 (*%*^e^)	32	*27*,*1*	378	*37*,*6*	122	*53*,*5*	154	*37*,*7*	68	*24*,*7*	283	*29*,*6*	32	*25*,*0*	93	*46*,*3*	254	*33*,*9*	908	*35*,*3*

^a^ Percentage of the caught fly population.

^b^ Percentage of dissected fly population.

^c^ Percentage of parous fly population.

^d^ Percentage of parous flies population with infective L3 larvae.

^e^ Percentage of development stages found in infected flies.

DS: dry season (October–March); RS: rainy season (April–September); L1: first-stage larvae; L2: second-stage larvae; L3: infective third-stage larvae.

### Comparison of *Simulium* biting rates and *Onchocerca* transmission potentials pre- and post-ivermectin distribution

**Fig 2:**
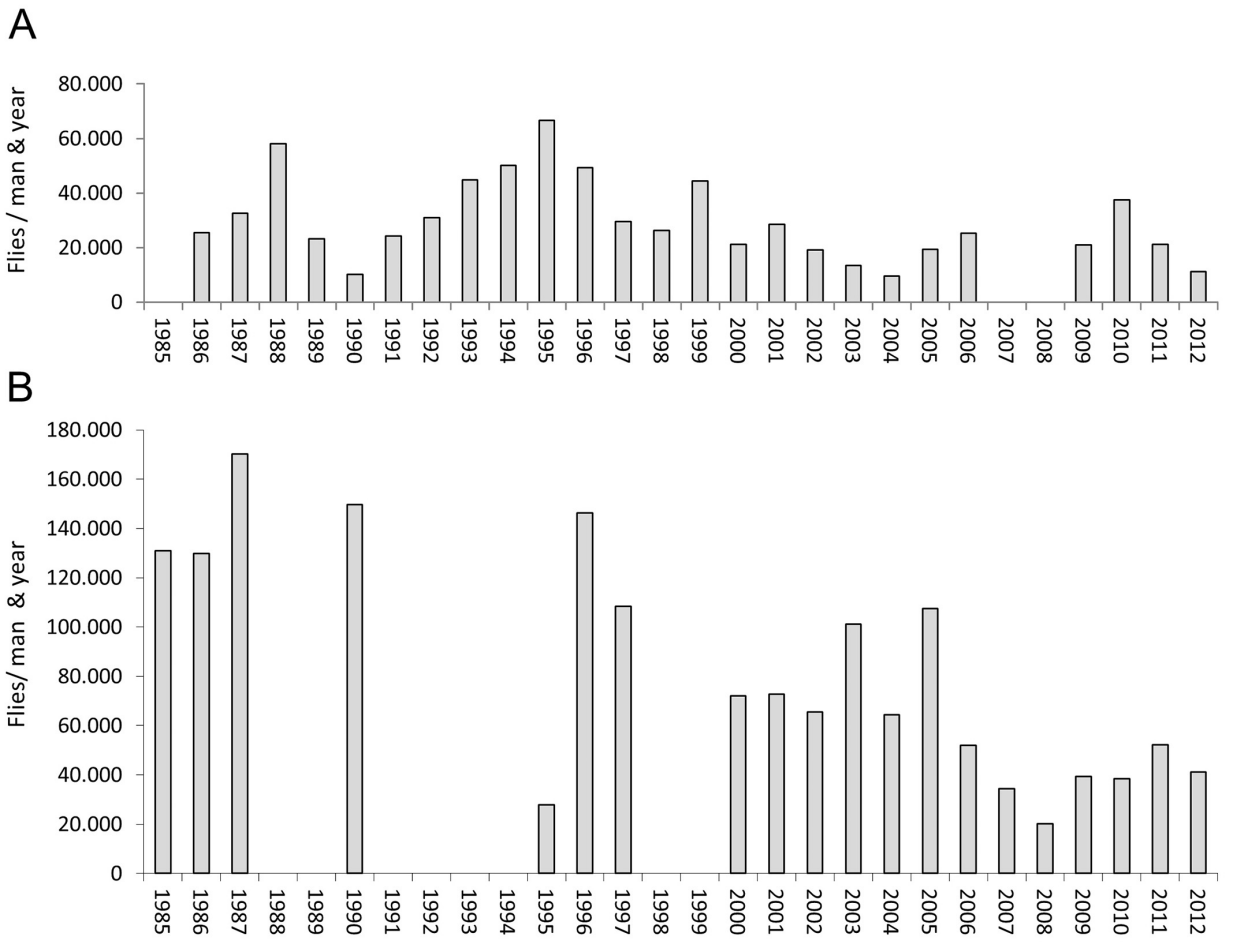
Annual biting rates. Annual biting rates of *Onchocerca* spp. from two onchocerciasis foci in North Cameroon. Each data point starts at the beginning of the rainy season (April) till the end of the dry season (March of the following year). Years with no data are left blank. **A** Soramboum, Vina du Nord. Epidemiological data prior to 1998 was published before and modified to fit this graph. Data from 1976 was taken from Touboro, 30 km further downstream. **B** Galim, Vina du Sud. Epidemiological data prior to 1997 was published before and modified to fit this graph.

**Fig 3:**
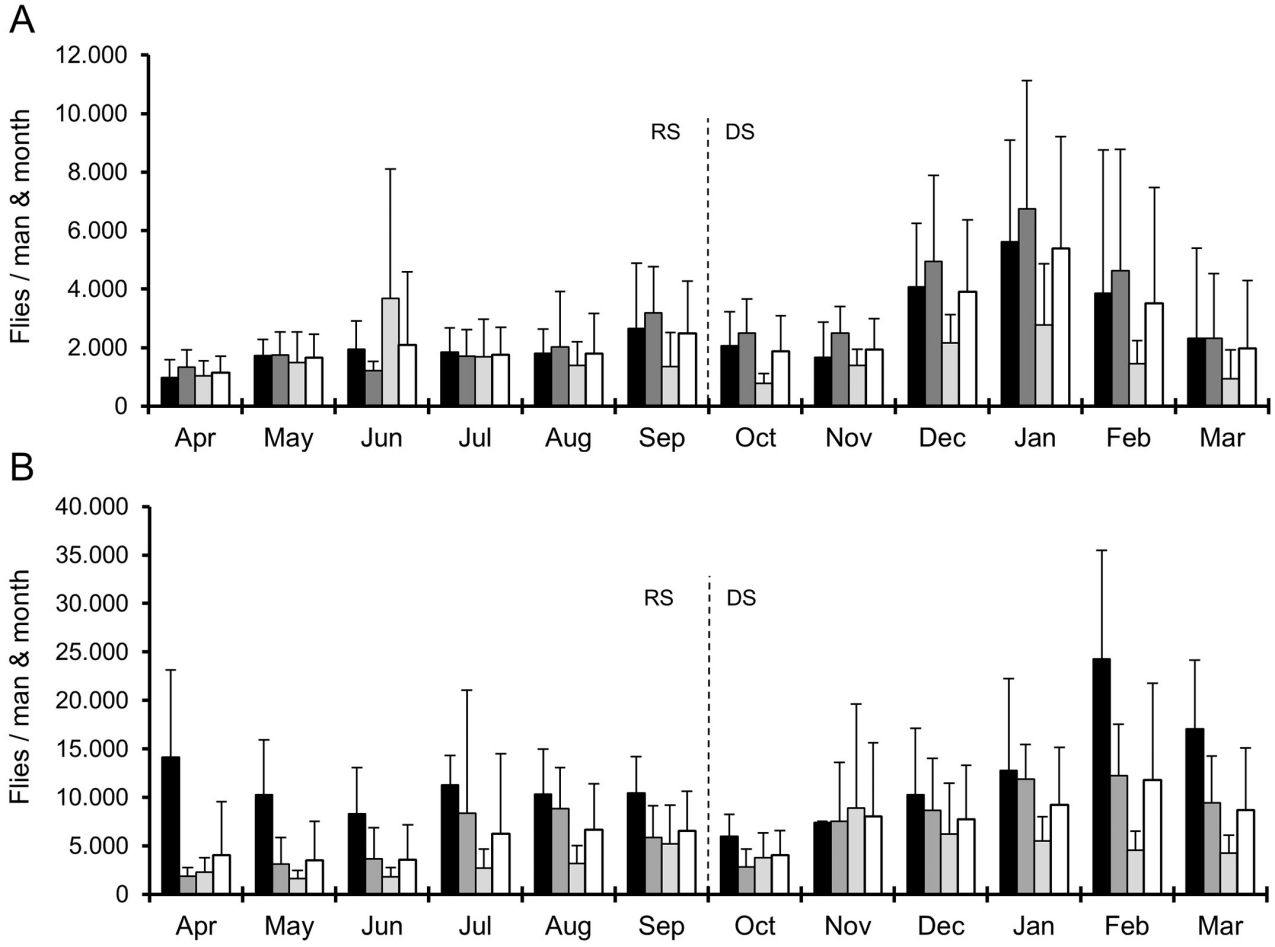
Monthly biting rates. Mean monthly biting rates of *Onchocerca* spp. from two onchocerciasis foci in North Cameroon. Whiskers show the standard deviation. Black bar: 1984–1993; dark grey bar: 1994–2003; light grey: 2004–2012; white bar: 1984–2012. RS: rainy season; DS: dry season. **A** Soramboum, Vina du Nord. **B** Galim, Vina du Sud.

Fig 2 shows the ABR for the two study sites starting prior to the distribution of ivermectin. With the exception of 1995 the ABR in Galim was higher than in Soramboum, on average by a factor of 4.26 (SD ±3.30; range: 0.42–14.67; n = 17). The yearly fluctuations were more pronounced in the Vina du Nord valley and followed a cyclical pattern (Fig 2A). In contrast, the ABR at the Vina du Sud fluctuated only mildly apart from intermittent dips, which reached previous levels in the following year (Fig 2B). An ongoing trend of lower biting frequencies was evident in Soramboum since 2002 (mean: 19,700 flies per person and year *vs*. 35,348 before) and in Galim since 2006 (mean: 39,628 flies per person and year *vs*. 103,564 before). In Soramboum the decline in biting rate occurred mainly in the dry season from October till March with only little changes during the rainy season (Fig 3A), whereas in Galim the highest decline was within the peak of the dry season and the peak of the rainy season from February till August (Fig 3B). In the same period of declining ABRs, the monthly infection rate of all L3-harboring flies of all *Onchocerca* spp. increased in Soramboum from 2.25% (1987–2001: 95%-CI: 0.45; n = 90) to 3.26% (2002–2012: 95%-CI: 0.53; n = 89), while it remained stable in Galim (1989–2005: 3.34%, 95%-CI: 0.57, n = 100 *vs*. 2006–2012: 3.19%, 95%-CI: 0.62, n = 65).

**Fig 4:**
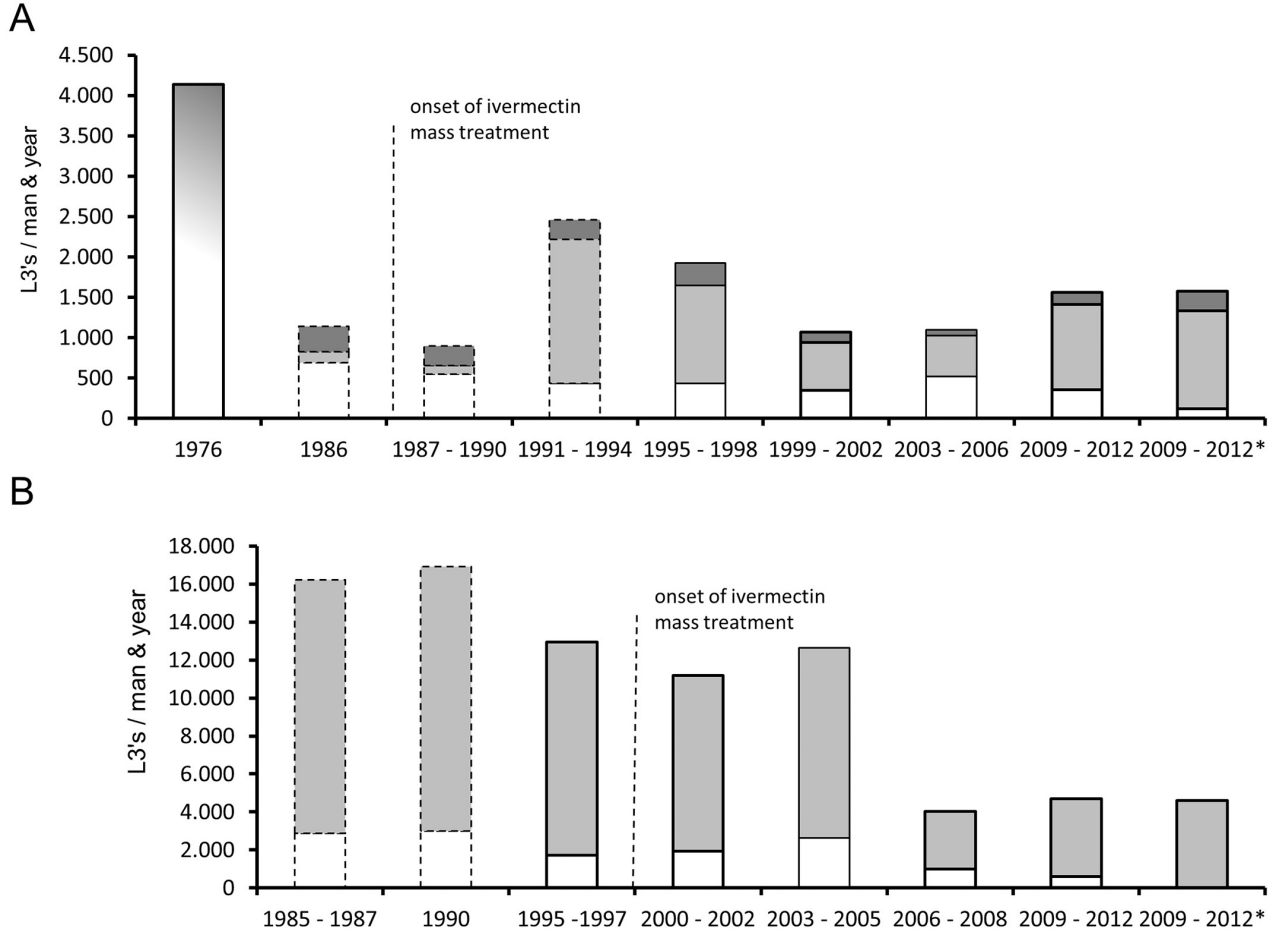
Annual transmission potentials. Annual transmission potentials of *Onchocerca* spp. from two onchocerciasis foci in North Cameroon. Data is given at the beginning of the rainy season (April) till the end of the dry season (March of the following year). Dark grey: *O*. *ramachandrini*; light grey: *O*. *ochengi*; white: *O*. *volvulus*. Insufficient fly dissection data from 1986, 1989–1992 in Soramboum, and from 1985–1987, 1990 and 1997 in Galim have been extrapolated. In 1976 (color transition of white, light grey to dark grey) no species discrimination was done. ATP with available MTP data ≥ 80% have borders in bold; those with data 80% > x ≥ 30% have normal borders, and those with data < 30% have borders with a dotted line. The dotted line marks the beginning of ivermectin mass treatments. The asterisk indicates the introduction of PCR-based methods for species discrimination in the vector. Prior to 1990, the L3 species was determined according to the following body length criteria: *O*. *volvulus* ≤ 750 μm; 750 μm < *O*. *ochengi* < 900 μm; *O*. *ramachandrini* ≥ 900 μm. **A** Soramboum, Vina du Nord. Epidemiological data prior to 1998 was published before and modified to fit this graph. Data from 1976 was taken from Touboro, 30 km further downstream. **B** Galim, Vina du Sud. Epidemiological data prior to 1997 was published before and modified to fit this graph.

A historic summary of the Annual Transmission Potentials over the last 36 years in Soramboum (Fig 4A) and 27 years in Galim (Fig 4B) illustrates the alterations in the ratio of animal-filariae and the human filaria *O*. *volvulus* in the vector. In Galim, annual filarial transmission rates remained high till 2006 (mean: 13,525 L3 per person and year, SD ±5334), when it dropped to 32.5% of previous levels (mean: 4395 L3 per person and year, SD ±2348; Fig 3B). In contrast, Soramboum experienced an increase of L3 transmission after the early years of ivermectin mass treatments, from an average ATP of 1045 ±438 L3 per person and year in 1987–88 to 2286 ±1338 in 1993–98, which later returned to former levels, *i*.*e*. an ATP of 1242 ±741 in 1999–2012 (Fig 4A), although the pre-ivermectin control ATP from the adjacent Touboro site was much higher (4140 L3 per person and year, Fig 3A).

### Species composition of the infective *Onchocerca* larvae from *Simulium damnosum s*.*l*.

**Fig 5:**
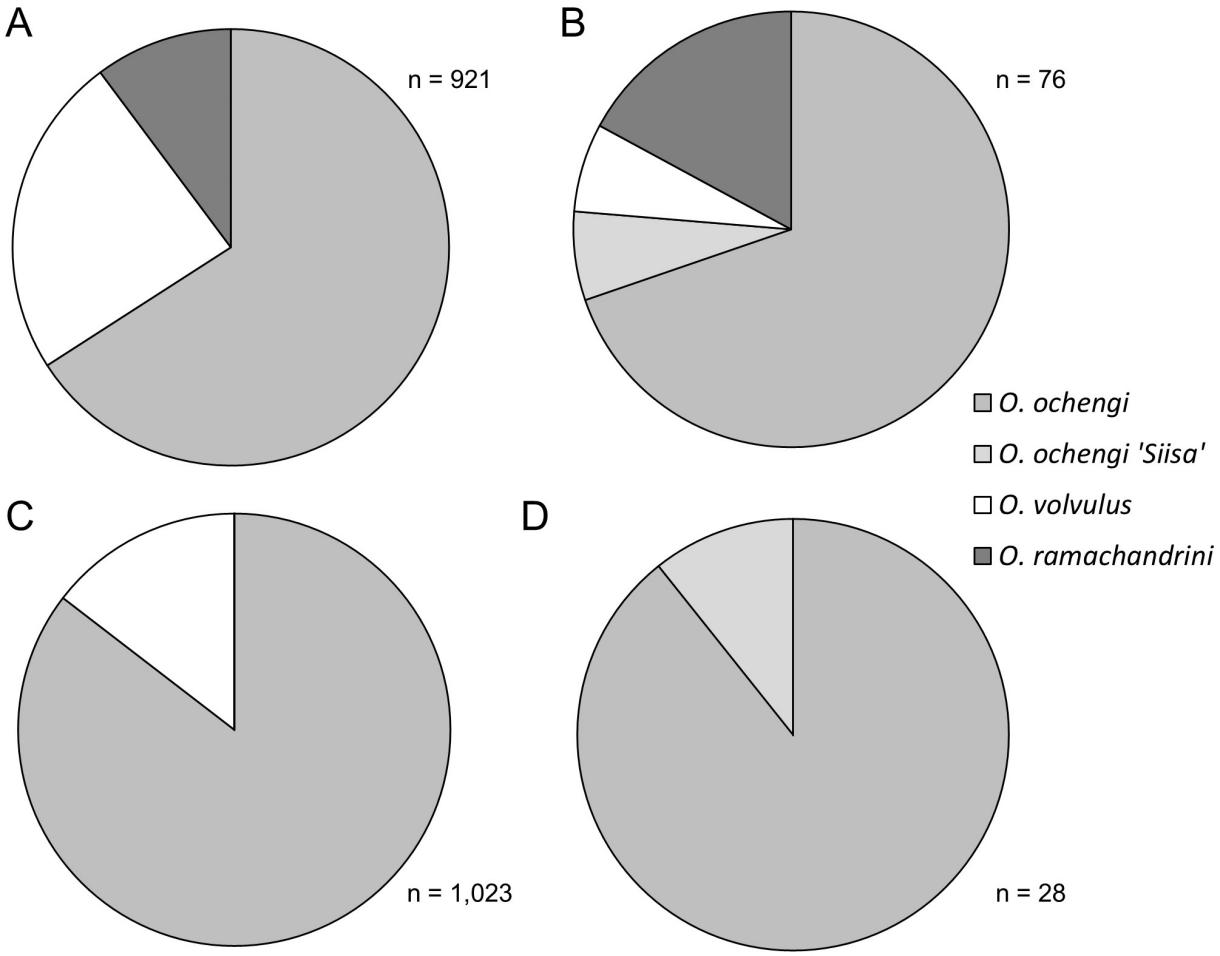
L3 species proportion. Species composition of infective third-stage larvae from *S*. *damnosum s*.*l*. at two foci in North Cameroon. White: *O*. *volvulus*; light grey: *O*. *ochengi* 'Siisa' type; medium grey: *O*. *ochengi*; dark grey: *O*. *ramachandrini*. **A-B** Soramboum, Vina du Nord. **C-D** Galim, Vina du Sud. Left side (A, C): morphological identification. Right side (B, D): PCR-based identification.

According to morphological classification the species composition of the L3 population in Soramboum from 2009 till 2012 was 23.9% *O*. *volvulus*, 65.9% *O*. *ochengi* and 10.2% *O*. *ramachandrini* (Fig 5A). In previous years the species composition of *O*. *volvulus*—*O*. *ochengi—O*. *ramachandrini* fluctuated from 60.7%–12.3%–27.0% (1987–88) to 22.3%–65.3%–10.2% (1993–99) and 40.5%–50.8%–8.7% (2000–06, Fig 4A). Correspondingly, the species were composed as follows in Galim: 11.3% *O*. *volvulus*, 88.7% *O*. *ochengi* (1995–96), 19.2%, 80.8% (2000–05) and 17.4%, 82.6% (2006–12, Fig 4B). No *O*. *ramachandrini* L3 were not found at all.

At the Vina du Nord site 96 isolated L3 (10.3% of all found) from 52 infected flies (7.2% of all dissected) were subjected to molecular identification, of which 76 (79.2%) PCR products were successfully sequenced. At Galim from the Vina du Sud site, 40 L3 (3.5% of all found) from 22 infected flies (2.8% of all dissected) provided 28 (70%) amplicons of *Onchocerca* spec. which could be successfully sequenced. The species composition of these L3 from Soramboum was 6.6% *O*. *volvulus*, 76.3% *O*. *ochengi* and 17.1% *O*. *ramachandrini* (Fig 5B), whereas in Galim only *O*. *ochengi* was found (Fig 5D). A recently discovered *O*. *ochengi* genotype called 'Siisa' [22,23,34] contributed to 8.6% and 10.7%, respectively, of the local *O*. *ochengi* L3 population in Soramboum and Galim (Fig 4B and 4D, respectively). In comparison with morphological classification (n = 71), 72.2% (13/18) of so-called *O*. *volvulus* in Soramboum were in fact *O*. *ochengi*, and 2.4% (1/41) of *O*. *ochengi* were *O*. *ramachandrini*. All examined *O*. *ramachandrini* (n = 12) were correctly identified. Hence, the respective effective ATP in Soramboum for the years 2009 to 2012 was 68, 221, 58 and 47 for *O*. *volvulus* (mean: 98): 773, 2503, 1388 and 475 for *O*. *ochengi* (mean: 1285) and 18, 392, 238 and 70 for *O*. *ramachandrini* (mean: 180). Accordingly, the adjusted species proportion of the L3 population for these years were on average 6.3% *O*. *volvulus*, 82.2% *O*. *ochengi* and 11.5% *O*. *ramachandrini* (Fig 4A, right side).

At the Vina du Sud site near Galim (n = 67) *O*. *volvulus* (0/46) and *O*. *ramachandrini* (0/0) have not been detected since the introduction of molecular methods for L3 species identification (Fig 5D), although there were morphologically identified specimens of *O*. *volvulus* (n = 149, 14.6% of total, Fig 5C). All morphologically classed *O*. *ochengi* (n = 21) were correctly identified. Hence, the whole L3 population in the observation period 2009–2012 consisted of *O*. *ochengi* (Fig 4B, right side) with an ATP of 5096, 4525, 6753 and 2046 (mean: 4605).

### L3 body length as a parameter in species identification

**Fig 6:**
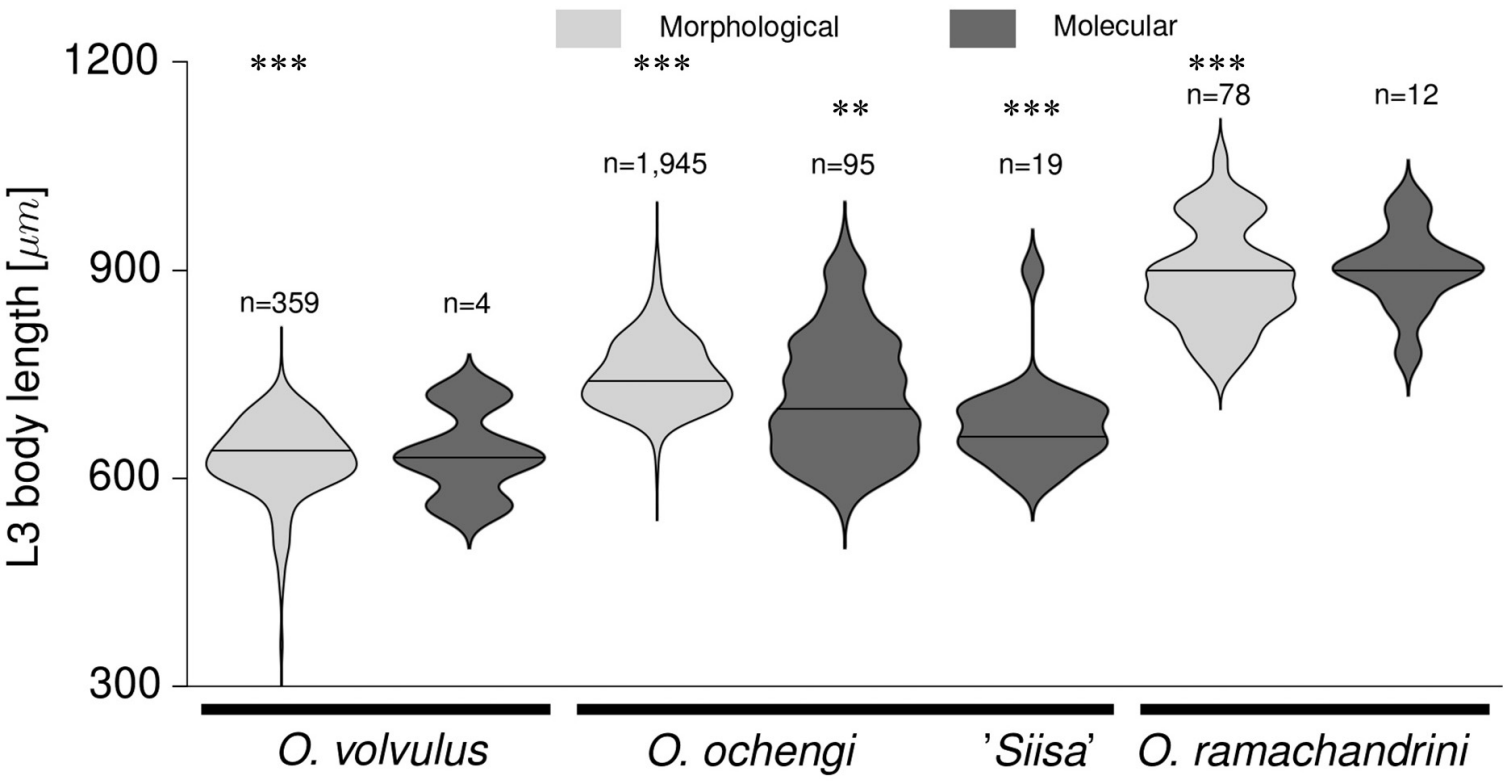
L3 body lengths. Violin plot of the L3 body lengths of identified *Onchocerca* spp. in *S*. *damnosum s*.*l*. from North Cameroon. Species identification is based on morphological (light grey) and molecular-genetic (dark grey) characteristics. Two genotypes of *O*. *ochengi*, *O*. *ochengi s*. *str*. and *O*. *ochengi* 'Siisa', are shown based on their mitochondrial clades. ***P < 0.001; **0.01 > *P* > 0.001; *P < 0.05.

In order to evaluate the reliability of body length as a characteristic trait that can be used for species discrimination of infective larvae, the body lengths of occurring *Onchocerca* spp. L3 in *S*. *damnosum s*.*l*. were compared with morphological and molecular identification methods (Fig 6). Whereas the inter-specific differences according to morphological criteria are significant (p < 0.001), no within-species length difference has been detected between morphological and molecular identification of *O*. *volvulus* and *O*. *ramachandrini*. A significant (p < 0.01) within-species difference has been found in the common genotype of *O*. *ochengi sensu stricto*, but with a low effect size (d = 0.598, n = 95); a significant difference (p < 0.001, d = 1.471, n = 19) was also found for the genotype *O*. *ochengi* 'Siisa'. Interestingly, a more than 4-times higher proportion of morphologically misidentified *O*. *volvulus* were *O*. *ochengi* 'Siisa' (25.4%; 15/59) than in the morphologically identified *O*. *ochengi* group (6.3%; 4/64). For the genotype *O*. *ochengi s*.*s*., this was vice versa (74.6%; 44/59 of misidentified *O*. *volvulus vs*. 92.2%; 59/64 of *O*. *ochengi* group).

## Discussion

This study represents a comprehensive 4-year dataset of transmission from sites in two onchocerciasis-endemic river basins and re-evaluates data collected up to 36 years ago. Whereas we can observe a break of the transmission cycle on the Adamawa plateau, the decline of parasite transmission seems to be halted in the focus of the Sudan savannah despite ongoing treatment intervention. The treatment intervention in this focus has passed the estimated life expectancy of the worm (10 to 15 years) almost by factor two. Conjected that the residual transmission does not stem from invading infected flies from other endemic foci by wind drift, the elimination of *O*. *volvulus* in previous hyperendemic foci in North Cameroon by yearly-given CDTI appears to be difficult, even though the actual risk of skin-lesions and blindness due to onchocerciasis is presumably very low.

Despite the fact that the Mectizan treatment campaigns on the mesoendemic Adamawa plateau have started about 10 years later, the transmission of the parasites there seems to be disrupted. One reason might be that the transmission rates in the past were partly overestimated due to misidentification of infective larval stages derived from animals. However, several studies including this one emphasize the adequate discriminative power of morphological characters for species delimitation, in particular the body length (Fig 6) and shape of head and tail [15,19,35]. It is thus very likely that at least a fraction of the L3 found were correctly identified as *O*. *volvulus*. However, if the endemicity of a region reaches hypoendemic levels in an area of intensive transmission of filariae of non-human origin, such as in this case, the reliability of microscopic examination is limited. For confirmation of interruptions of *O*. *volvulus* transmission, molecular methods like PCR are necessary.

### Decline of the *Simulium damnosum* biting rates of humans

Since 2006 there is a steady trend of lower ABRs, in particular at the Vina du Sud river, where biting rates before were with only one exception above 60,000 per man and year (Fig 2B). The vector transmission of filarial stages have also dropped during this time (Fig 4B), but to a lesser extent in the Sudan savannah (Fig 4A) due to a concomitant gain of the infection rate by bovine filariae. The reason for this vector decline could be the result of decreased availability of breeding sites or food for the aquatic *Simulium* larvae, and hence a drop in population size. A distinct increase of endoparasitic mermithids in human-biting nulliparous flies was evident at the Vina du Sud breeding sites (S1 table). It is, however, unlikely that these mermithids or other *Simulium* parasites are the main drivers for the massive decline in biting rates of recent years. A reduced longevity of adult flies was not observed, when comparing the current parous rate with those of flies at baseline [24]. Furthermore, a continuous rise in the pool of potential blood hosts, both human and livestock, may also contribute to lower individual biting frequencies. The regional impact of climate change cannot be excluded, either, although the water delivery of the investigated rivers have not changed drastically until 1980 [26].

### Endemicity of *Onchocerca volvulus* in North Cameroon

The longitudinal monitoring in the Vina du Nord valley indicates that the average transmission of *O*. *volvulus* remained around 500 L3 per man and year for 20 years after the onset of ivermectin mass treatments (Fig 4A). This seems to contradict the reduction of onchocerciasis-positive patients in the region as a result of control strategies with ivermectin [12]. One reason could be that there is a variable degree of misidentification of *O*. *volvulus* L3. In the most recent monitored years 2009–2012, when molecular detection methods were used, the degree of morphological misidentification was 72%. However, earlier epidemiological data from the Sudan savannah [25] showed ATP above 4000 L3 per man and year (Fig 4A, left side). Even though no differentiation of the species had been undertaken at that time, the proportion of animal-filariae in *S*. *damnosum s*.*l*. were likely low due to the lack of cattle as potential blood hosts [for explanation see 36]. Hence, only filariae from the warthog could have been co-transmitted, and the infection rate of the vector with *O*. *ramachandrini* has not changed considerably during the observation period. Another theory states that the regulation of parasite transmission may be density-dependent instead of linear. That means the effective reproductive ratio of filarial worms equals one even though the basic reproductive ratio is much higher. In the Vina du Nord river basin, Renz [25] compared the prevalence of onchocerciasis and burden of microfilariae with the L3 infection rate in the vector and found no linear correlation, but rather a dependency of fly infection rate with prevalence in the human population instead of the community's microfilarial load (mff/mg). A density-dependent mechanism has already been shown for the parasite acquisition in cattle when inoculated with infective larvae of *O*. *ochengi* [37,38].

The observed seasonal variations of the entomological parameters match well with baseline data from the Vina du Nord river [25], including the number of infective flies with L3. Nonetheless, the *O*. *volvulus* ATP has drastically reduced to 3.5% of the baseline value meaning that the majority of infective flies now carry filariae of animal origin. Additionally, the number of L3 per infective fly decreased moderately (from 3.2 to 1.8). The low but stable transmission level of *O*. *volvulus* could mean that the threshold for maintaining endemicity is perhaps lower than current mathematical models predict (ATP ≥ 100 in West Africa [39,40], but also ATP ≥ 54 in Central America [41]).

### Molecular *vs*. morphologic L3 species identification

Even though molecular techniques of identification give higher accuracy, they are less suitable for high throughput analysis due to limitations of time, cost-effectiveness and local infrastructure. They are nonetheless very useful for the detection of unknown strains and species of filarial nematodes in vector and host, such as *Onchocerca ochengi* 'Siisa' [22,23,34]. Experimental infection studies from Togo [42] and Mali [43], where *O*. *ochengi* microfilariae were inoculated by the vector from infected cattle, revealed shorter L3 body lengths (Togo: 680 μm, 540–680; Mali: 647 μm, 540–810) than our observations (740 μm, 600–940) and previous studies from Cameroon [44]. These values, however, lie in proximity to the measurements for *O*. *ochengi* 'Siisa' (660 μm, 600–900) and may thus represent or morphologically resemble this strain. Ultimately the evolutionary relationships of *Onchocerca* parasites in humans, cattle and game animals can be compared and tested with genetic markers by generating phylogenetic trees [22,34].

### Influence of closely-related animal filariae for the transmission of river blindness

Besides the climatic disparities of the two foci, which is mainly a result of different altitudes, they share similar conditions for their respective black fly populations. One major difference, however, is the disproportionately higher cattle stock density on the Adamawa plateau compared to the situation in the Sudan savannah (Fig 1). The cattle to human ratio around the Galim focus is approx. 10:1, whereas in the Soramboum focus it ranges between 1:1 and 2:1, and was even lower in previous years, because nomadic cattle were not allowed to enter the Vina du Nord basin until 1975, and the local villagers had not kept any livestock animals, either. Nowadays, an increasing number of vagrant Bororo herdsmen arrive with their herds of zebu cattle and become settled. The inherent difference in livestock density is both culturally inherited (migrating pastoralists of the North vs. settled cattle farmers of the South) and due to biologic conditions (water and food scarcity during the dry season in the Sudan savannah; absence of tsetse flies on the Adamawa plateau, which transmit bovine trypanosomiasis). Invading *O*. *ochengi* L3 elicit a humoral immune reaction in humans, which cross-reacts with *O*. *volvulus* L3 antigens, thereby reducing transmission success [19]. The protective effect of populations under repeated antigen exposure is called premunition and well known for malaria and other infectious diseases [45,46]. On the Adamawa plateau this effect seems to be strong enough to prevent or at least complicate the regional endemicity of *O*. *volvulus*. The advent of nomadic herdsmen and their cattle herds in the Vina du Nord valley is congruent with an increased transmission of animal-borne filariae, in particular *O*. *ochengi* (Fig 4A). This sudden jump of animal-filariae in the vector population implies the diversion of large quantities of local *S*. *damnosum s*.*l*. to take their blood meal from cattle, thereby reducing the vector pool for humans [16]. This phenomenon has been termed zooprophylaxis and acts also as a protective trait against onchocerciasis transmission [15,20].

The important question is how the low but stable rate of onchocerciasis transmission in the Sudan savannah can be further curbed or completely stopped. Altogether, five molecularly identified *O*. *volvulus* L3 from two infective flies (3.85% of total, 95% CI: 0.47–13.21%) were found in the dry season of 2010 (February) and the rainy season of 2011 (June). Since yearly CDTI application rounds are given at the end of July, the late time point of occurrence after ivermectin treatment may hint to an incipient reconstitution of skin microfilariae in humans infected with *O*. *volvulus* 12 months prior. This would be a strong argument for the continuation or even temporary intensification of the ivermectin control program [10,36,47]. However, current political instabilities in adjacent countries and the exclusion of certain patient groups in treatment intervention programs, like nomadic people, illegal immigrants and refugees, could impede the long-term success of such measures. Ongoing monitoring of vector transmission is therefore crucial for health policy in onchocerciasis-endemic countries.

## Supporting Information

S1 Table Infection rate of female nulliparous flies and aquatic larvae of *Simulium damnosum s.l*. at two different sites in Northern Cameroon. Those infection percentages from mermithids, fungus and malpighian nematodes are given from nulliparous female flies and aquatic *Simulium* larvae, respectively, and those from planidium larvae from all flies dissected.
(PDF)
