# Supplementary material for: Ongoing Transmission of Onchocerca volvulus after 25 Years of Annual Ivermectin Mass Treatments in the Vina du Nord River Valley, in North Cameroon
Source: PLoS Negl Trop Dis. 2016 Feb 29;10(2):e0004392. doi: 10.1371/journal.pntd.0004392 (PMC4771805; doi:10.1371/journal.pntd.0004392)
Supplement: S1 Table — Those infection percentages from mermithids, fungus and malpighian nematodes are given from nulliparous female flies and aquatic Simulium larvae, respectively, and those from planidium larvae from all flies dissected. (PDF) [file pntd.0004392.s001.pdf]

Table S1: Infection rate of female nulliparous flies and aquatic larvae of *Simulium damnosum s.l.* at two different sites in Northern Cameroon.

Location		DS 2009/10		RS 2010		DS 2010/11		RS 2011		DS 2011/12		RS 2012		Total	
Galim,	mean monthly biting rate	4.907		1.660		3.900		2.459		6.224		1.874		3.504	
Vina du Sud	flies dissected	1.783		947		1.014		962		2.168		773		7.647	
	nulliparous (%)	734	*41,2*	432	*45,6*	369	*36,4*	361	*37,5*	715	*33,0*	352	*45,5*	2.963	*38,7*
	with mermithids (%)	37	*5,0*	19	*4,4*	2	*0,5*	17	*4,7*	34	*4,8*	8	*2,3*	117	*3,9*
	with fungus round form (%)	0	*0*	1	*0,2*	0	*0*	0	*0*	1	*0,1*	0	*0*	2	*0,1*
	with malpighian nematode (%)	5	*0,7*	1	*0,2*	4	*1,1*	3	*0,8*	5	*0,7*	0	*0*	18	*0,6*
	with planidium larva (%)	0	*0*	3	*0,3*	1	*0,1*	0	*0*	0	*0*	0	*0*	4	*0,1*
	larvae dissected													250	
	with mermithids (%)													14	*5,6*
	with fungus round form (%)													1	*0,4*
	with malpighian nematode (%)													2	*0,8*
Soramboum,	mean monthly biting rate	1.844		3.662		2.579		1.738		1.803		711		2.056	
Vina du Nord	flies dissected	2.141		4.236		2.636		1.393		1.128		561		12.095	
	nulliparous (%)	1.085	*50,7*	768	*18,1*	771	*29,3*	176	*12,6*	299	*26,5*	124	*22,1*	3.223	*26,6*
	with mermithids (%)	1	*0,1*	0	*0*	1	*0,1*	0	*0*	0	*0*	0	*0*	2	*0,1*
	with fungus round form (%)	4	*0,4*	1	*0,1*	0	*0*	1	*0,6*	0	*0*	0	*0,0*	6	*0,2*
	with malpighian nematode (%)	1	*0,1*	3	*0,4*	2	*0,3*	2	*1,1*	0	*0*	2	*1,6*	10	*0,3*
	with planidium larva (%)	0	*0*	6	*0,1*	1	*0,04*	0	*0*	0	*0*	0	*0*	7	*0,1*
				RS 1995		DS1995/96		RS 1996		DS1996/97				Total	
Galim,	mean monthly biting rate			1.552		3.084		11.145		13.230				7.253	
Vina du Sud	flies dissected			574		800		2.564		2.050	*26,5*			5.988	
	nulliparous (%)			210	*36,6*	247	*30,9*	534	*20,8*	544	*48,2*			1.535	*25,6*
	with mermithids (%)			1	*0,5*	0	*0*	5	*0,9*	3	*0,6*			9	*0,6*
	with fungus round form (%)			0	*0*	0	*0*	0	*0*	0	*0*			0	*0*
	with malpighian nematode (%)			0	*0*	0	*0*	0	*0*	0	*0*			0	*0*
	with planidium larva (%)			0	*0*	0	*0*	0	*0*	0	*0*			0	*0*
